# Comparison between Manual and Automated Methods for Ki-67 Immunoexpression Quantification in Ameloblastomas

**DOI:** 10.1155/2016/7486989

**Published:** 2016-10-24

**Authors:** Rogelio González-González, Nelly Molina-Frechero, Ramón G. Carreón-Burciaga, Sandra López-Verdín, Carlos Robles-Bonilla, Vanesa Pereira-Prado, Ronell Bologna-Molina

**Affiliations:** ^1^Department of Research, School of Dentistry, Universidad Juárez del Estado de Durango, Durango, DGO, Mexico; ^2^Department of Health Care, Universidad Autónoma Metropolitana, Unidad Xochimilco, Mexico City, Mexico; ^3^Microbiology and Pathology Department, Dentistry Research Institute, Health Sciences University Center, Universidad de Guadalajara, Guadalajara, JAL, Mexico; ^4^School of Health Sciences, Universidad Anahuac Norte, Lomas Anáhuac, Huixquilucan, MEX, Mexico; ^5^Department of Molecular Pathology, School of Dentistry, Universidad de la República, Uruguay, Montevideo, Uruguay

## Abstract

Ameloblastoma is a common and unpredictable odontogenic tumor with high relapse rates. Several studies assessing the proliferative capacity of these neoplasms have been published, mainly using the protein Ki-67. Cell counts must be completed to determine the cell proliferation rate. Multiple methods have been developed for this purpose. The most widely used method is the labeling index, which has undergone changes over time to better facilitate cell counting. Here, we compared manual cell counting methods with automated cell counting (ImmunoRatio) to determine the relative effectiveness of these methods. The results suggest that ImmunoRatio, a free software tool, may be highly advantageous and provide results similar to manual cell counting methods when used with the appropriate calibration. However, ImmunoRatio has flaws that may affect the labeling index results. Therefore, this automated cell counting method must be supplemented with manual cell counting methods.

## 1. Introduction

Odontogenic tumors are a heterogeneous set of lesions, including nonneoplastic proliferations and benign and malignant neoplasms [1, 2]. Ameloblastoma (AM) is considered to be the second most common odontogenic tumor, accounting for more than 30% of odontogenic neoplasms in the majority of epidemiological studies [3, 4]. AM is typically classified as unicystic (UA), solid/multicystic (SMA), desmoplastic, and peripheral, and ameloblastic carcinoma is its malignant counterpart. Each of these neoplasms exhibits histological variations that may affect their clinical behavior [4]. The expression of the Ki-67 protein is strictly associated with cell proliferation. Ki-67 is present during all active cell cycle phases (G1-M) and absent during the quiescent phase (G0). Ki-67 is an excellent marker for determining the growth fraction of cell populations, and Ki-67 immunoexpression is an auxiliary tool to define the prognosis of various tumors [5]. Tumors with rapid cell proliferation can indicate a poorer prognosis when compared with tumors with low cell proliferation. Cell counts must be performed to determine the growth fraction of cell populations, and the labeling index is the most frequently used method [6]. We proposed a simple change to this method in 2008 [7], including the use of a photomicrograph analysis software suite that increased cell counting accuracy and decreased rater fatigue. However, this method does not enable the rater to mark the counted cells. In 2015 [8], a variation of this cell counting method using the ImageJ software was proposed. This software increased cell counting accuracy and enabled the marking of counted cells, thus improving the comparison between datasets. However, cell counting is time-consuming, despite the high degree of accuracy. The ImmunoRatio (http://153.1.200.58:8080/immunoratio/) is a free web application for automated image analysis that decreases the time required for cell counting. ImmunoRatio has two variant methods of cell count: basic and advanced methods; the basic method (IRB) is characterized by its not modifying software parameters, while the advanced method (IRA) is characterized by its modifying software parameters, by means of blank field correction image, source image scale, and other. This software can be used directly on the website or through the plugin of ImageJ, with similar characteristics: plugin with basic method (IRpB) and plugin with advanced method (IRpA). This method has yet to be assessed for AMs. Thus, it remains still unknown if this software can provide results similar to conventional cell counting methods. In the present study, we compared two manual cell counting methods with the automated ImmunoRatio method in the most common AM variants to determine if these methods can be used interchangeably for this type of odontogenic tumor.

## 2. Materials and Methods 

Thirty-four AMs tissue samples (15 UA and 19 SMA) fixed in buffered formalin and embedded in paraffin blocks were obtained from the Laboratory of Molecular Pathology of the University of the Republic in Uruguay (UDELAR). Two 3 *μ*m sections were cut from each block using a microtome. The first section was stained with hematoxylin and eosin to confirm the histopathological diagnosis according to the World Health Organization (WHO) classification [4]. The diagnoses using the hematoxylin and eosin stained samples were completed by two experienced pathologists. The second set of tissue sections were mounted on silanized slides and immunohistochemical staining was performed according to previously described methods [9]. The primary antibody used was anti-Ki-67 (Clone MIB-1, Monoclonal Mouse, Anti-Human, IgG1, Dako Corp., Carpinteria, CA, USA). Cervical lymph nodes were used as a positive control, and the negative control staining was performed without primary antibody. All tissue sections processed without primary antibody lacked positive immunostaining.

Modified labeling index (manual scoring) and digital manual scoring/ImageJ cell counting (DM scoring) were compared. The manual scoring and DM scoring methods for Ki-67 immunostaining were performed according to Bologna-Molina et al. [7] and Carreón-Burciaga et al. [8], respectively.

To validate the cell counts using these methods, each AM case was evaluated in triplicate by three experienced pathologists blinded to the histopathological diagnosis. The results were considered valid if the kappa coefficient of interrater agreement was substantial to almost perfect (0.61–1.00) (Table 1).

### 2.1. Manual Scoring

**Figure 1:**
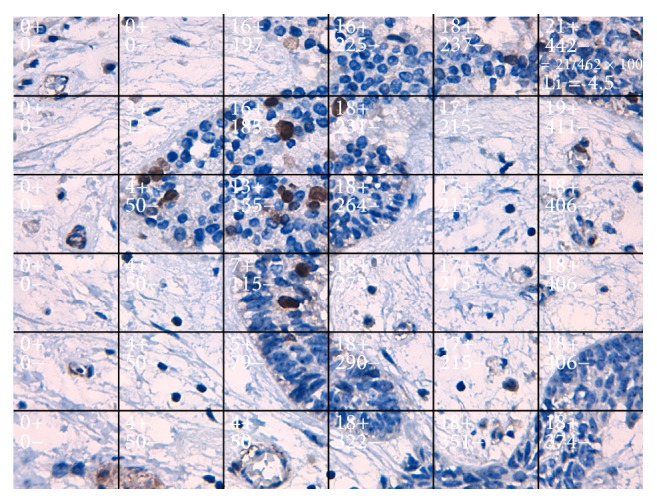
Photomicrograph of a solid ameloblastoma specimen at 400x magnification. Each box in this photomicrograph represents a cumulative number of nuclei with positive and negative Ki-67 staining. The last field shows the labeling index, which indicates the number of Ki-67-positive cells (method proposed by Bologna-Molina et al. [7]).

In accordance with Bologna-Molina et al. [7], five photomicrographs were chosen for this method, and the growth fraction for each AM case was determined (labeling index) (Figure 1).

### 2.2. DM Scoring

**Figure 2:**
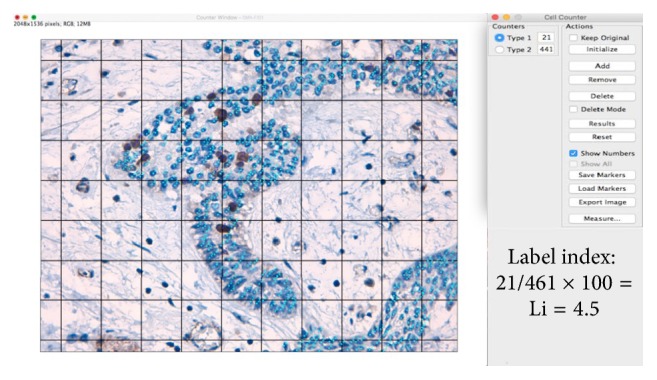
Photomicrograph of a solid ameloblastoma specimen at 400x magnification. This photomicrograph represents the manual cell counting method (DM scoring) using the Grid and Cell Counter plugins. The Grid was constructed with an area-per-point of 17,046 pixels^∧^2. The light blue dots indicate nuclei negative for DAB or Ki-67 staining. The dark blue nuclei indicate positive DAB and Ki-67 staining (ImageJ, method proposed by Carreón-Burciaga et al. [8]).

The DM scoring method is a variation of the manual scoring method. DM scoring was completed as described by Carreón-Burciaga et al. [8], and the Grid and Cell Counter plugins for ImageJ were used (https://imagej.nih.gov/ij/) (Figure 2).

### 2.3. Automated ImmunoRatio Method

**Figure 3:**
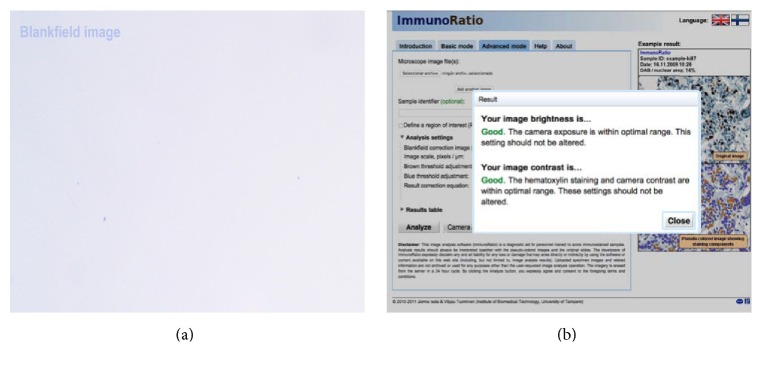
The blank field image used to evaluate the photomicrographs of each ameloblastoma case. (a) The blank field image from a microscope with a digital camera (MOTIC BA-210 and MOTICAM 5.0). (b) The blank field brightness and contrast test indicating the brightness and contrast quality. This procedure is highly recommended for the advanced ImmunoRatio methods (IRA scoring and IRpA scoring). The test was conducted at the following web address: http://153.1.200.58:8080/immunoratio/.

ImmunoRatio is a free web application for automated image analysis that is based on separation and deconvolution imaging, nuclear thresholding, particle segmentation, filtration, and calculation of the nuclear area ratio using diaminobenzidine (DAB) immunostaining. The nuclear area was determined using two methods: the basic method (IRB scoring) without any adjustment and the advanced method (IRA scoring) with color correction according to the protocol of Tuominen et al. [10]. As suggested in the protocol, we also used a blank field image that was validated using the Camera Adjustment Wizard test according to the ImmunoRatio requirements (Figures 3(a) and 3(b)). The basic and advanced ImmunoRatio methods (IRpB scoring and IRpA scoring, resp.) can also be used directly with ImageJ by applying the ImmunoRatio plugin (http://jvsmicroscope.uta.fi/sites/default/files/software/immunoratio-plugin/index.html). The nuclear area analysis was performed according to the online ImmunoRatio method. As proposed by Bologna-Molina et al. [7], we obtained the labeling index of the nuclear area for both online and ImageJ ImmunoRatio methods using automated cell counting in five fields of view at 40x magnification [7].

## 3. Results 

### 3.1. Manual Scoring and DM Scoring

The mean cell counts and labeling indexes by variant of AMs obtained by each method are outlined in Table 1. Manual scoring and DM scoring: these methods showed little variation in mean cell count in AMs (manual scoring, Li = 12.35, SD ± 7.60; DM scoring, Li = 12.35, SD ± 7.61) and a substantial kappa coefficient for interrater agreement (manual scoring versus DM scoring) (Figures 1 and 2). Cell count times ranged from approximately 35 to 50 minutes for each case evaluated.

### 3.2. ImmunoRatio

Cell count performed by advanced and basic methods by variants of AMs is shown in Table 2.

**Figure 4:**
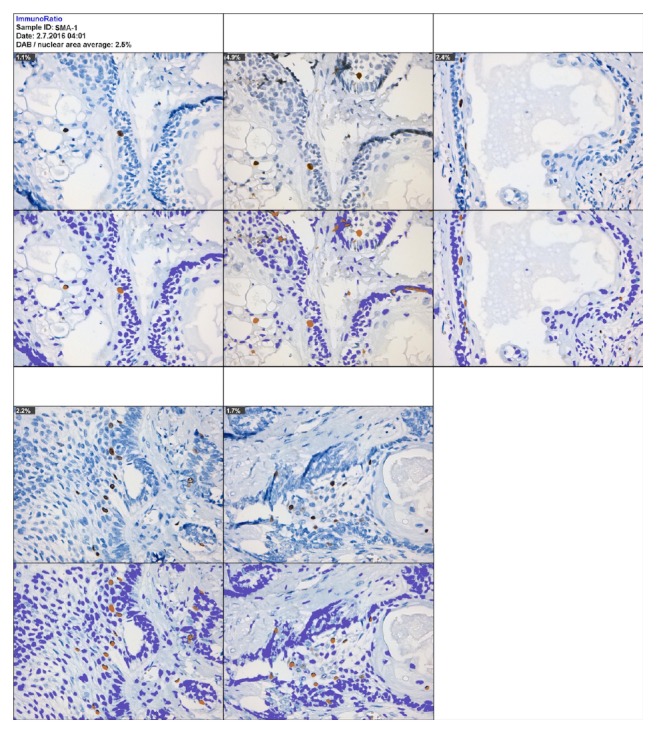
Photomicrograph of a solid ameloblastoma specimen at 400x magnification using the advanced ImmunoRatio method. The online advanced ImmunoRatio method (IRA scoring) was performed at http://153.1.200.58:8080/immunoratio/ using the previously validated blank field image and the appropriate changes for cell counting. These changes are shown in Figure 5(a).

**Figure 5:**
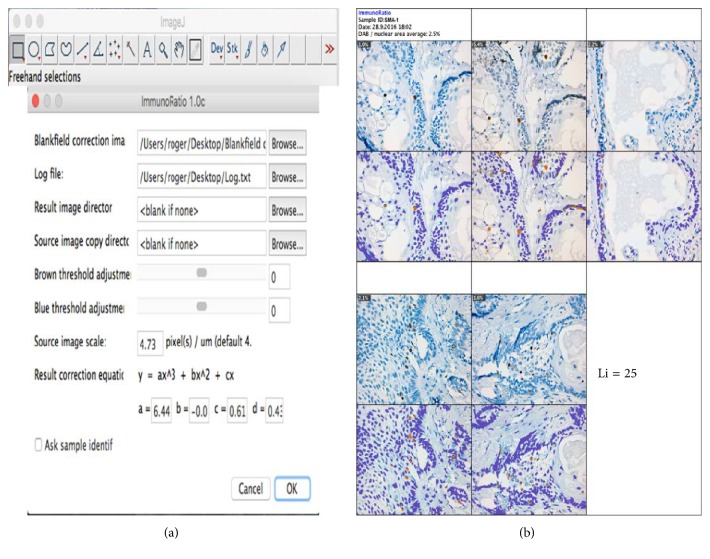
The advanced ImmunoRatio method using the ImageJ ImmunoRatio plugin. IRpA scoring: (a) changes introduced to the advanced ImmunoRatio method were obtained from the online advanced ImmunoRatio method. (b) Photomicrographs of a solid ameloblastoma specimen at 400x magnification. This photomicrograph represents the advanced ImmunoRatio method for determining the labeling index. This method was modified according to Bologna-Molina et al. [7]. The results are from five photomicrographs. Each original photomicrograph is shown in the top panel, and the ImmunoRatio results used to generate the informative pictorial montage are shown in the bottom panel.

**Figure 6:**
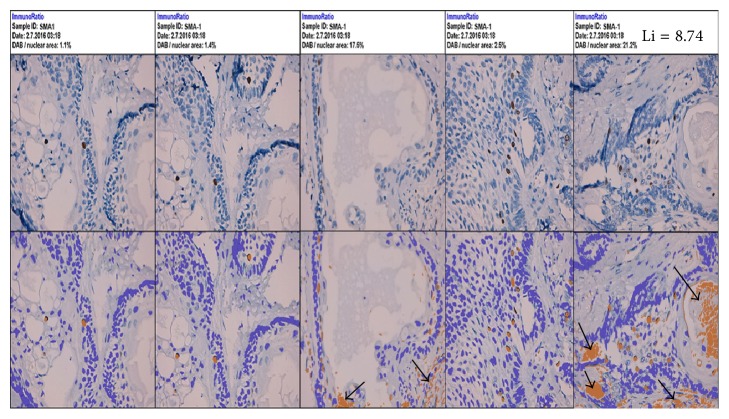
Photomicrographs of a solid ameloblastoma specimen at 400x magnification using the online basic ImmunoRatio method (IRB scoring). Arrowheads indicate the false positive zones in the pictorial montage.

**Figure 7:**
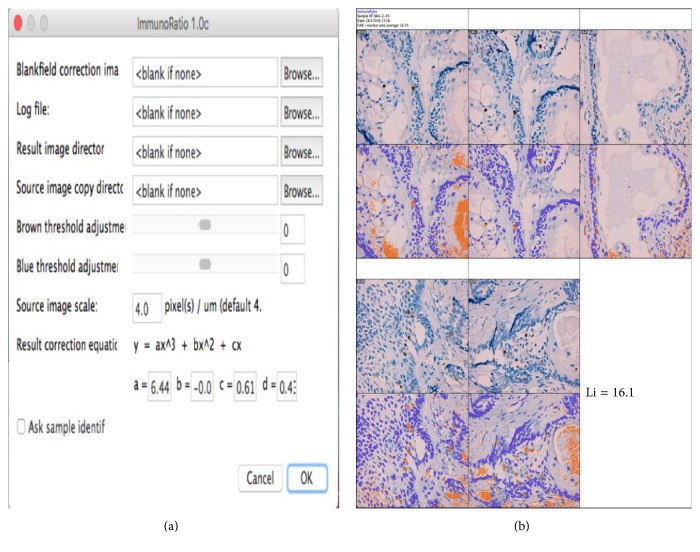
Photomicrographs of a solid ameloblastoma specimen at 400x magnification using the basic ImmunoRatio method with the ImageJ plugin (IRpB scoring). (a) Table of modifications that were not performed. (b) Cell counts for the original photomicrographs (top panel) and the pictorial montage (bottom panel) with false positives similar to those shown in Figure 6.

The advanced method: ImmunoRatio web and the plugin of ImmunoRatio ImageJ show little difference at cell count in AMs (IRA scoring, Li = 12.10, SD ± 9.88; IRpA scoring, Li = 12.10, SD ± 9.87) and substantial kappa in each variant of AM, resulting in almost perfect kappa coefficients (IRA scoring versus IRpA scoring). Cell count times ranged from approximately 1 to 2 minutes for each case evaluated (Figures 4 and 5 and Table 3). Basic method: the online and ImageJ plugin basic ImmunoRatio methods showed minimal variation in mean cell count in AMs (IRB scoring, Li = 12.56, SD ± 8.29; IRpB scoring, Li = 12.53, SD ± 6.16) and had slight kappa coefficients (IRB scoring versus IRpB scoring) with cell count times ranging approximately from 1 to 2 minutes for each case evaluated (Figures 6 and 7 and Table 3).

### 3.3. Kappa Coefficients

The comparison between the manual scoring, DM scoring, and ImmunoRatio (advanced and basic) methods showed kappa coefficients ranging from slight to null (Table 3).

### 3.4. Pearson's Correlation

Pearson's correlation coefficient was significant for all of the methods used in our study. The results in Table 4 show a strong positive correlation between manual scoring and DM scoring and between the advanced ImmunoRatio methods (IRA scoring and IRpA scoring). A moderate positive correlation was found between the basic and advanced ImmunoRatio methods. The correlation between the manual method and the basic or advanced ImmunoRatio methods was weak.

## 4. Discussion 

The Ki-67 antigen is a cell proliferation marker that has been widely used for prognosis and to determine the proliferation capacity of tumor cells in various tumors [11]. Being able to establish the proliferation index of AMs is of fundamental importance towards the evaluation of its biological behavior: local invasion and tumorous recurrence [12]. Because of this, the use of antigen Ki-67 as a marker for cell proliferation is an auxiliary complement of great relevance towards the identification and prognosis of odontogenic tumors [12].

The labeling index method [6] and its modified version [7] are widely used to assess the growth fraction in AMs. Although highly reproducible, these cell counting methods are time-consuming, tedious, and exhausting. The modified version of the labeling index method with ImageJ [8] (DM scoring) enables the rater to mark the counted cells, thereby ensuring increased reproducibility and decreased fatigue during counting; however, this is still a manual method. Our present findings show that the manual methods used to determine the labeling index are reproducible, and the results correlate with the proliferation capacity of tumor cells in AM [6, 9, 13–16]. However, several factors may affect the analysis of Ki-67, including the quality of immunostaining, cell count area selection, and rater expertise [17]. The significant kappa coefficients and highly positive Pearson's correlation coefficients calculated in our present study (Tables 3 and 4) are likely due to the triplicate image analysis and the cell counting expertise of the raters. Multiple types of image analysis software have been developed for quantitative pathology [17], making it possible to decrease cell counting time and rater fatigue. Studies with nuclear markers indicate that computer-assisted labeling index assessments can provide results similar to those obtained with manual methods [17]. ImmunoRatio is a free and automated method for image analysis and labeled cell counting [18]. This method has produced suitable results in mammary and neuroendocrine tumors [10, 17, 18]. In AM, our findings indicate that cell counts obtained with the advanced ImmunoRatio method (IRA scoring and IRpA scoring) were somewhat similar to manual counting methods, although we observed small kappa values (<0.39) and weak Pearson's correlation coefficients (<0.5). Nevertheless, the percentage difference between conventional labeling indexes (manual scoring and DM scoring) and advanced ImmunoRatio (IRA scoring and IRpA scoring) was minimal (<0.5%), suggesting that advanced ImmunoRatio is a suitable method for labeling index assessments in AMs. Our data corroborates the findings published by Remes et al. [17], who reported that ImmunoRatio is a suitable method to assess the labeling index in neuroendocrine tumors. Importantly, adequate photomicrographs with optimal contrast and brightness and an adequate blank field image that meets both brightness and contrast quality standards must be used to obtain results with minimal differences between manual methods and the advanced ImmunoRatio method. In addition, the use of the basic ImmunoRatio method (IRB scoring and IRpB scoring) may be suitable when software calibration is not required. Our analysis of the basic ImmunoRatio method showed minimal percentage differences (<0.5%) between the advanced ImmunoRatio method and the manual methods (IRB scoring, IRpB scoring versus IRA scoring, IRpA scoring, manual scoring and DM scoring). This suggests that ImmunoRatio methods may be used interchangeably. Notably, false positives may result from the lack of adequate calibration, thus considerably changing the labeling index data (Figure 6).

Using the ImmunoRatio method to determine the labeling index has advantages over manual methods. First, it is a sophisticated image analysis method that enables the calculation of multiple parameters quickly, accurately, and reliably. Computer-assisted assessments are not affected by rater errors, and the results from photomicrographs are analyzed in exactly the same way. The automated method requires no peer calibration or prior experience in cell counting, and the cell counting time is minimal with no chance of rater fatigue. These are key factors that may affect cell counting when using conventional methods. ImmunoRatio generates an informative pictorial montage that can be easily evaluated [18]; furthermore, it is a free, open-access web application that can be used offline with the ImageJ plugin [18]. However, this automated method also has some disadvantages, including the requirement of photomicrographs with adequate quality and contrast and an appropriate immunohistochemical technique. Moreover, this method may be affected by the intensity of Ki-67 staining, which varies according to the cell cycle phase [17]. This method is also unable to differentiate tumor cells from nontumor cells with DAB-positive nuclei, which may cause errors in the labeling index results.

In conclusion, the advanced ImmunoRatio method is a powerful cell counting tool that enables the observation and assessment of the number of positive and negative cells by generating pictorial montages. Analyses using the advanced ImmunoRatio method are highly reproducible and more objective than conventional methods and much less time-consuming. Furthermore, the results are similar to conventional methods, although adequate software calibration and previous training are required. Additional studies on AMs are required to assess if ImmunoRatio is a fast and efficient cell counting method that yields results similar to conventional methods. At present, ImmunoRatio should not be used as a substitute cell counting method. Instead, labeling indexes determined by using the advanced ImmunoRatio method should be compared with those obtained from manual methods.

The use of advanced ImmunoRatio may be as efficient as the evaluation of label index by conventional methods, given that optimal software adjustments are made by an experienced observer. In this present study, the use of this software is established for the first time on AMs. This is relevant to medical practice since it enables quick results at cell counting, which demonstrates that it is a valuable auxiliary means towards establishing prognosis to this kind of tumors.

## Figures and Tables

**Table 1:** Cell counts used to determine the labeling index were obtained with manual methods: manual scoring and DM scoring. The total tumor cell count per ameloblastoma variant and the kappa coefficient obtained for each rater are shown. *κ* = (scoring 1 versus 2, 1 versus 3, and 2 versus 3). The kappa coefficient ranged from substantial to almost perfect with *p* = 0.000 in all cases. ^*∗*^Digital manual scoring/ImageJ scoring.

AM	*n* = 34, (100)	Total cell count ± SD	Manual scoring (1) ± SD	Manual scoring (2) ± SD	Manual scoring (3) ± SD	*κ* = (scoring 1 versus 2)	*κ* = (scoring 1 versus 3)	*κ* = (scoring 2 versus 3)	*p*
UA	15, (44.1)	1226 ± 547.82	14.29 ± 7.43	14.28 ± 7.44	14.26 ± 7.44	0.858	0.789	0.856	0.000
SMA	19, (55.8)	1839 ± 867.68	10.81 ± 7.58	10.8 ± 7.58	10.8 ± 7.58	0.888	0.833	0.723	0.000

AM	*n* = 34, (100)	Total cell count ± SD	^*∗*^DM scoring (1) ± SD	^*∗*^DM scoring (2) ± SD	^*∗*^DM scoring (3) ± SD	*κ* = (scoring 1 versus 2)	*κ* = (scoring 1 versus 3)	*κ* = (scoring 2 versus 3)	*p*

UA	15, (44.1)	1226 ± 547.78	14.32 ± 7.42	14.5 ± 7.5	14.5 ± 7.51	0.994	0.669	0.928	0.000
SMA	19, (55.8)	1839 ± 867.72	10.78 ± 7.57	10.65 ± 7.53	10.62 ± 7.53	0.944	0.888	0.723	0.000

**Table 2:** Automatic method used to determine label index with basic and advanced ImmunoRatio. The table shows the total of tumorous cells obtained from ameloblastoma by the use of techniques of ImmunoRatio; the kappa index obtained for each range is also shown. The kappa index ranged from substantial to almost perfect for all the advanced methods of ImmunoRatio in all cases. *κ* = (scoring 1 versus 2) and *p* = 0.000. For the basic methods of ImmunoRatio, the Kappa index was moderate *κ* = (scoring 1 versus 2) and without statistical significance for the UA. The advanced methods: IRA scoring (ImmunoRatio advanced scoring) and IRpA scoring (ImmunoRatio plugin advanced scoring). Basic methods: IRB scoring (ImmunoRatio basic scoring), IRpB (ImmunoRatio plugin basic scoring). IRA scoring and IRB scoring were used with the webpage of ImmunoRatio. IRpA and IRpB were used with the program ImageJ and the plugin of ImmunoRatio.

AM	*n* = 34, (100%)	Total cell count ± SD	IRA scoring (1) ± SD	IRpA scoring (2) ± SD	*κ* = (scoring 1 versus 2)	*p*
UA	15, (44.1)	N/A	15.69 ± 13.29	15.69 ± 13.30	0.856	0.000
SMA	19, (55.8)	N/A	9.25 ± 4.68	9.25 ± 4.67	0.726	0.000

AM	*n* = 34, (100%)	Total cell count ± SD	IRB scoring (1) ± SD	IRpB scoring (2) ± SD	*κ* = (scoring 1 versus 2)	*p*

UA	15, (44.1)	N/A	12.16 ± 7.86	13.32 ± 8.0	0.054	0.057
SMA	19, (55.8)	N/A	12.87 ± 8.81	11.91 ± 4.26	0.050	0.000

**Table 3:** The cell counting methods used to determine the labeling index. The kappa coefficient was obtained by comparing each method. The manual (manual scoring and DM scoring) and advanced ImmunoRatio (IRA scoring and IRpA scoring) methods showed substantial kappa coefficients. Italic text indicate substantial kappa coefficient and bold text indicates a significance level of *p* < 0.05.

Technique versus	Manual scoring	DM scoring	IRA scoring	IRpA scoring	IRB scoring	IRpB scoring
Manual scoring	N/A	***0.698***	**0.026**	**0.024**	**0.026**	−0.003
DM scoring	***0.698***	N/A	−0.002	−0.004	0.023	−0.002
IRA scoring	**0.026**	−0.002	N/A	***0.788***	−0.003	−0.005
IRpA scoring	**0.024**	−0.004	***0.788***	N/A	−0.003	−0.003
IRB scoring	**0.026**	0.023	−0.003	−0.003	N/A	**0.055**
IRpB scoring	−0.003	−0.002	−0.005	−0.003	**0.055**	N/A

**Table 4:** The cell counting methods used to determine the labeling index. Pearson's correlation coefficient was obtained by comparing each method. The manual (manual scoring and DM scoring) and advanced ImmunoRatio methods (IRA scoring and IRpA scoring) had nearly perfect positive Pearson's correlation coefficients, whereas the basic ImmunoRatio methods (IRB scoring and IRpB scoring) had moderate positive correlation coefficients when compared with the advanced ImmunoRatio method. Italic text indicates moderate to strong Pearson's correlation and bold text indicates a significance level of *p* < 0.05.

Technique versus	Manual scoring	DM scoring	IRA scoring	IRpA scoring	IRB scoring	IRpB scoring
Manual scoring	N/A	***r*** = ***1.000***	*r* = 0.300	*r* = 0.299	**r** = 0.341	*r* = 0.301
DM scoring	***r*** = ***1.000***	N/A	*r* = 0.303	*r* = 0.302	**r** = 0.342	*r* = 0.304
IRA scoring	*r* = 0.300	*r* = 0.303	*N/A*	***r*** = ***1.000***	***r*** = ***0.602***	***r*** = ***0.686***
IRpA scoring	*r* = 0.299	*r* = 0.302	***r*** = ***1.000***	N/A	***r*** = ***0.601***	***r*** = ***0.685***
IRB scoring	**r** = 0.341	**r** = 0.342	***r*** = ***0.602***	***r*** = ***0.601***	N/A	***r*** = ***0.775***
IRpB scoring	*r* = 0.301	*r* = 0.304	***r*** = ***0.686***	***r*** = ***0.685***	***r*** = ***0.775***	N/A

## References

[1] Fernandes A. M., Duarte E. C. B., Pimenta F. J. G. S. (2005). Odontogenic tumors: a study of 340 cases in a Brazilian population. *Journal of Oral Pathology and Medicine*.

[2] Ramos G. D. O., Porto J. C., Vieira D. S. C., Siqueira F. M., Rivero E. R. C. (2014). Odontogenic tumors: a 14-year retrospective study in Santa Catarina, Brazil. *Brazilian Oral Research*.

[3] Fregnani E. R., da Cruz Perez D. E., de Almeida O. P., Kowalski L. P., Soares F. A., de Abreu Alves F. (2010). Clinicopathological study and treatment outcomes of 121 cases of ameloblastomas. *International Journal of Oral and Maxillofacial Surgery*.

[4] Barnes L., Eveson J. W., Reichart P., Sidransky D. (2005). *World Health Organization Classification of Tumours: Pathology and Genetics of Head and Neck Tumors*.

[5] Scholzen T., Gerdes J. (2000). The Ki-67 protein: from the known and the unknown. *Journal of Cellular Physiology*.

[6] Meer S., Galpin J. S., Altini M., Coleman H., Ali H. (2003). Proliferating cell nuclear antigen and Ki67 immunoreactivity in ameloblastomas. *Oral Surgery, Oral Medicine, Oral Pathology, Oral Radiology, and Endodontics*.

[7] Bologna-Molina R., Damián-Matsumura P., Molina-Frechero N. (2011). An easy cell counting method for immunohistochemistry that does not use an image analysis program. *Histopathology*.

[8] Carreón-Burciaga R. G., González-González R., Molina-Frechero N., Bologna-Molina R. (2015). Immunoexpression of Ki-67, MCM2, and MCM3 in ameloblastoma and ameloblastic carcinoma and their correlations with clinical and histopathological patterns. *Disease Markers*.

[9] Bologna-Molina R., Mosqueda-Taylor A., Lopez-Corella E. (2008). Syndecan-1 (CD138) and Ki-67 expression in different subtypes of ameloblastomas. *Oral Oncology*.

[10] Tuominen V. J., Ruotoistenmäki S., Viitanen A., Jumppanen M., Isola J. (2010). ImmunoRatio: a publicly available web application for quantitative image analysis of estrogen receptor (ER), progesterone receptor (PR), and Ki-67. *Breast Cancer Research*.

[11] Gerdes J., Schwab U., Lemke H., Stein H. (1983). Production of a mouse monoclonal antibody reactive with a human nuclear antigen associated with cell proliferation. *International Journal of Cancer*.

[12] Abdel-Aziz A., Amin M. M. (2012). EGFR, CD10 and proliferation marker Ki67 expression in ameloblastoma: possible role in local recurrence. *Diagnostic Pathology*.

[13] Olimid D. A., Florescu A. M., Cernea D. (2014). The evaluation of p16 and ki67 immunoexpression in ameloblastomas. *Romanian Journal of Morphology and Embryology*.

[14] Metgud R., Gupta K. (2013). Expression of cell cycle and apoptosis-related proteins in ameloblastoma and keratocystic odontogenic tumor. *Annals of Diagnostic Pathology*.

[15] Nafarzadeh S., Seyedmajidi M., Jafari S., Bijani A., Rostami-Sarokolaei A. (2013). A comparative study of PCNA and Ki-67 expression in dental follicle, dentigerous cyst, unicystic ameloblastoma and ameloblastoma. *International Journal of Molecular and Cellular Medicine*.

[16] González-González R., Molina-Frechero N., Damian-Matsumura P., Salazar-Rodriguez S., Bologna-Molina R. (2015). Immunohistochemical expression of Survivin and its relationship with cell apoptosis and proliferation in ameloblastomas. *Disease Markers*.

[17] Remes S. M., Tuominen V. J., Helin H., Isola J., Arola J. (2012). Grading of neuroendocrine tumors with Ki-67 requires high-quality assessment practices. *The American Journal of Surgical Pathology*.

[18] Vijayashree R., Aruthra P., Rao K. R. (2015). A comparison of manual and automated methods of quantitation of oestrogen/progesterone receptor expression in breast carcinoma. *Journal of Clinical and Diagnostic Research*.

